# *In vitro* clinical trials: the future of cell-based profiling

**DOI:** 10.3389/fphar.2014.00121

**Published:** 2014-05-28

**Authors:** Nathan T. Ross, Christopher J. Wilson

**Affiliations:** ^1^Developmental and Molecular Pathways, Novartis Institute for Biomedical ResearchCambridge, MA, USA; ^2^Neuroscience, Novartis Institute for Biomedical ResearchCambridge, MA, USA

**Keywords:** *in vitro* clinical trials, cell line profiling, cell panels, genetically defined models, iPS derived cell models, high throughput screening

## Abstract

The drug discovery process classically revolves around a set of biochemical and cellular assays to drive potency optimization and structural-activity relationship models. Layered on top of these concepts is the inclusion of molecular features that affect final drug use, things like: bioavailability, toxicity, stability, solubility, formulation, route of administration, etc. Paradoxically, most drugs entering clinical trials are only tested in a handful of human genetic backgrounds before they are given to people. Here we review efforts and opine on the use of large scale *in vitro* cellular and *in vivo* models that attempt to model human disease and include diversity found in the human genetic population. Because hundreds to thousands of individual assays are needed to scratch the surface of human genetic diversity, sophisticated high throughput automation technologies or pooling and deconvolution strategies are required. Characterization of each model needs to be extensive to enable non-biased informatics based modeling. Such approaches will enable deep understanding of genetic to pharmacological response and result in new methods for patient stratification in the clinic. Oncology medicines and cancer genetics have been paving the way for these approaches and we expect to see continued expansion to other fields such as immunology and neuroscience.

## INTRODUCTION

When a drug discovery project starts, a project team must conduct an important thought experiment: if a perfect molecule that meets all of the team’s criteria was suddenly available, what patients would be selected and what clinical assays would be used to demonstrate efficacy. In genetically well-defined diseases this can be a conceptually straight-forward task. For example in monogenic, recessively inherited diseases, like sickle cell anemia or spinal muscle atrophy, patients are readily identifiable by symptoms in the clinic and confirmed genetically. A homogenous population of patients provides the best chance of achieving a high signal to noise readout in the clinical trial. This is because all the patients should be similar in their molecular pathology and patients treated with a targeted drug should, in an ideal scenario, be obligate responders.

Cancer, at a fundamental level, is more heterogeneous, but patient selection strategies have made tremendous strides in the last decade. An example is the use of Gleevec and other Abl kinase inhibitors in chronic myelogenous leukemia (CML) patients that harbor the Philadelphia Chromosome translocation creating a BCR-Abl gain of function fusion protein. Prior to the use of Abl inhibitors, approximately 10,000 people in the US died each year due to CML; that number has dropped to less than 500 people per year since the introduction of these drugs to the market. Other examples of therapies “targeted” to selected cancer types are abundant and include B-RAF, C-KIT, p53-MDM2, c-MET, JAK1, and EGFR. In many cases these therapies lead to a remission, not a cure, and this is likely a reflection of the genetic heterogeneity of the original tumor and a result of the subsequent selective pressure under drug treatment that enable resistant cells to continue to grow. Ultimately, this will lead to drug resistance. New next gen sequencing (NGS) technologies are tremendously impacting the field’s understanding of the spectrum of mutations and underlying heterogeneity in a tumor.

Other “common” diseases, like Alzheimer’s disease, Type II Diabetes, and Schizophrenia are thought to be at least partially driven by common genetic variants. The genetics are complicated but the prevailing theory is that small changes in gene regulation, likely at the level of mRNA, slightly predispose an individual for a disease. As subtle genetic variations accumulate in an individual, that person’s risk of developing the disease also increases. Due to this, genetic stratification in common disease populations is extremely difficult. Nonetheless, common variant research has identified examples where genetic testing of patients can be used to select patients for clinical trials. These include people who are homozygous for a common variant in APOE, ApoE4, whom are 10 times more likely to develop Alzheimer’s Disease relative to persons not harboring the ApoE4 variant. An excellent review by Plenge et al. (2013) draws connections between common variants and disease understanding; it outlines strategies for using genome wide association studies and other human genetic data to select drug targets and stratify patients.

**FIGURE 1:**
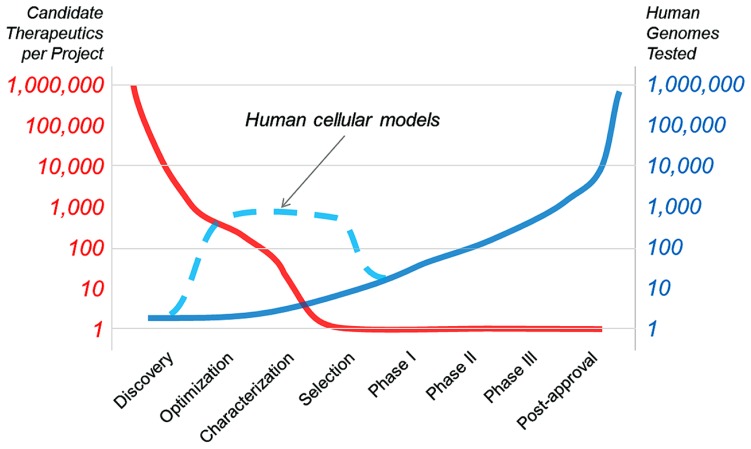
**The goal of “*in vitro* clinical trials” is to use genetically characterized cell panels in an effort to reduce the number of potential therapies dropping out of clinical development before entering clinical trials and to better inform patient stratification**.

Cancer therapeutics have already benefitted from the use of genetically defined human cell panels. This approach should be applicable to monogenic neurological disorders and eventually to other indications where predictive cellular models can be generated. The goal of “*in vitro* clinical trials” will be to change the arc of drug development, from a paradigm where many therapies are dropped between optimization and candidate selection (**Figure 1**). Human cellular models will help bridge this gap (as illustrated in **Figure 1**, dashed line), speeding the development of new drugs and our understanding of human disease. In this Technology Report, we aim to highlight past examples of cell panel screens and point to future applications of the approach that will extend past cancer to rapidly evolving areas like neurobiology.

## GENETIC STRATIFICATION WITH CANCER CELL LINES – HISTORICAL PERSPECTIVE

In the late 1980’s the national cancer institute (NCI) began profiling a set of approximately 60 human cancer cell lines in what became known as the NCI-60 panel (Shoemaker, 2006). The profiling platform was the first to enable researchers with drug candidates to find out whether their molecule of interest displayed any selectivity towards certain cancers and to focus a molecule in indications based on pre-clinical data. What was remarkable about the effort was the realization that cancer cell lines could capture some of the diversity of patient cancers and that with brute force effort, one could emulate a small scale clinical trial resulting in some predictive value. The NCI-60 was also pioneering from a technical perspective, establishing rigorous methods for genetically distinguishing cell lines from each other and enabling methods for large scale data analysis across many cell lines and compound treatments so that overlapping bioactivity between small molecules could be easily observed. Some of the major findings that have emerged from the NCI-60 effort include the link between TGFα-PE38 and EGFR expression, halichondrin B being identified as a microtubule inhibitor, and bortezomib/Velcade^TM^ as a proteasome inhibitor. Additionally, the recognition that cells could pump out a small molecule via the multidrug resistance pump (e.g., MDR and PgP) and that this could be modeled across cell lines could only be appreciated in the context of a panel of cell lines; the NCI-60 effort was truly pioneering in this approach.

The evolution of approaches to measure genomic chromosomal aberrations led Garraway and Sellers to look for genetic amplifications across the NCI-60 panel of cell lines as a potential means to identify new oncogenic drivers (Garraway et al., 2005). This work led to the discovery of microphthalmia-associated transcription factor (MITF) as a lineage specific oncogenic transcription factor in melanoma. Using single nucleotide polymorphism (SNP) arrays, the NCI-60 cell lines, spanning nine different cancer lineages, were assayed for chromosomal alterations. Hierarchical clustering was used to organize the cell lines by copy number alteration and the observation was made that the cell lines largely clustered by cancer type leading the authors to postulate that the chromosomal alterations driving these clusters might contain lineage-specific cancer genes. One of the most striking clusters was chromosome 3 (3p13–3p14) in the melanoma lineage. In their subsequent studies the authors used gain-of-function and loss-of-function approaches to demonstrate that MITF is capable of cooperating with mutant BRAF to transform normal cells and that MITF knockdown causes loss of viability in MITF copy-gain melanoma cells. This study served as another example of the early use of cell panels and their utility in leading to significant biological discovery that would not have been possible with individual disease models.

The approach taken by Garraway and Sellers was further extended in a report by Solit et al. (2006) where the genetic composition of the NCI-60 cell lines was compared against compound activity profiles, allowing the authors to link MEK inhibition to BRAF mutant melanoma. Subsequent studies have led to new applications such as that of Neve et al. (2006). Using a panel of 51 breast cancer cell lines and Trastuzumab, they identified an important relationship between HER2 amplification status and response to Trastuzumab treatment (Neve et al., 2006).

Pushing the cancer cell panel approach further, McDermott et al. (2007) established a panel of 500 cancer cell lines and tested 14 kinase inhibitors across this panel of lines. Their data confirmed the established relationships between EGFR, HER2, MET, and BRAF inhibitors and cell lines with the respective mutant gene. As had been observed previously at the NCI, the cell panel approach allowed for compounds with similar bioactivity profiles to be grouped together, revealing previously unappreciated biological activity and/or compound polypharmacology. In a subsequent manuscript McDermott et al. (2008) expanded their cell panel to 602 cell lines and detailed the relationships between ALK inhibitors and ALK mutant cancers. More recently several teams have established large cell line panels of > 500 cancer cell lines and have published profiling results of a sub-set of the compounds screened. These efforts include those of MGH/Sanger group (Garnett et al., 2012) and the Broad/Novartis Cancer Cell Line Encyclopedia effort (Barretina et al., 2012). There are several good reviews on the development of these large scale cancer cell panels (Shoemaker, 2006; Sharma et al., 2010; Caponigro and Sellers, 2011) and their application to patient selection and pharmacological compound analysis.

## TECHNICAL CONSIDERATIONS FOR THE SUCCESSFUL APPLICATION OF CELL PANELS AND OPPORTUNITIES FOR IMPROVEMENT

Many of the same challenges that were faced by the NCI-60 team in the 1980’s still exist today. First among these is the quality control (QC) of cell line identity. As has been the case since the inception of the cancer cell line screening panel, knowing the absolute identity of each cell line screened is critical for all subsequent analyses. Early on, HeLa cell contamination was mitigated by cytogenetic testing of cell lines via chromosome banding (Shoemaker, 2006). This process was also able to tease out cell lines derived from a common origin, adding an important layer of information for future data analyses. Since this time, technology in the field has evolved, starting with restriction length polymorphism analysis, on to DNA fingerprinting, then spectral karyotyping, and most recently, short tandem repeat (STR) or SNP testing. Other practices for QC, important in the past and still relevant today, are mycoplasma testing of all cell lines at each iteration of a panel screen and limiting the experiment to a common media type for tissue culture. In recent years, high-precision automation and the use of 1536-well format cellular assays have vastly increased the throughput at which cancer cell line panels can be screened. While this is powerful in light of the volume of data generated, which can lead to more interesting correlations of compound-cell activity, the requirement for appropriate QC measures will be even more important.

Much like the technology for cell line QC, assay readouts have also advanced over the last 30 years. NCI’s pioneering work led to the development of a high throughput screening assay using sulphorhodamine B as a read out (Shoemaker, 2006). This assay proved robust and conveniently included a fixation step before reading, which was highly desirable before the advent of modern automation equipment. Other early examples of large scale screening used 96-well plates and a cell-fixation assay with a fluorescent nucleic acid stain (McDermott et al., 2007). These protocols were modernized as part of the Cancer Cell Line Encyclopedia (CCLE) effort, when industrial-scale screening systems were brought to bear on the cell profiling challenge. In the published CCLE screen Novartis adapted the assay to 1536-well format and used Cell Titer Glo (Promega) as a one-step luminescent assay for cell viability (Barretina et al., 2012). However, even in this modern interpretation of the NCI-60 assay some methods remain the same, such as fixed seeding densities and standard media for all cell lines tested.

While proper QC is essential for yielding good data, selection of the ideal metrics for comparing compound response profiles across cell line panels is central to turning good data into impactful data. Today’s myriad methods for genetically characterizing cell lines offer many possibilities for dissecting the details of compound activity and cell-line responsiveness. Early efforts in this area include COMPARE for NCI-60 compound profiles (Shoemaker, 2006) and CellMiner (Shankavaram et al., 2009) for compound-genetic feature comparisons. Beyond the genetic feature correlations, there are a number of other parameters that can add to the complexity, such as dose-response curve IC50 (or crossing point), curve inflection point, activity area (or area under the curve), and maximal activity, frequently being utilized (Barretina et al., 2012). Most recently, Fallahi-Sichani et al. (2013) have demonstrated that incorporation of the concentration-response curve slope, area under the curve, and maximal effect can also yield insights into the mechanism of cell-death and also identify cell-to-cell variability in drug response.

Further insight can be gained by collecting more complex genetic information. Recently Jaffe et al. (2013) conducted global chromatin mass spectrometry profiling of 115 cancer cell lines. A common internal standard allowed for comparison of relative methylation and acetylation levels of all lysine residues on histone H3 between cell lines. This led to the observation that a set of cell lines had increased histone 3 lysine 36 dimethylation and that these lines contained either a t(4;14) translocation or a previously unknown coding mutation in the histone 3 lysine 36 methyltransferase NSD2, which was later shown to be an activating mutation. The t(4;14) translocation is known to drive high expression of NSD2, providing further support for the overlapping histone methylation profile of these cell lines. Increasing the depth of characterization of cancer cell lines will likely lead to further novel observations such as those described here.

## MOVING BEYOND CANCER CELL LINES

Even at the earliest stages of the NCI-60 cell panel project, the importance of transitioning from cancer cell lines to animal models of cancer was recognized. Limitations to cancer cell lines include inherent biases towards specific cell signaling and growth pathways that favor growth in cell culture. For example PI3Kalpha, RAS, and BRaf mutations are well represented in cancer cell lines. However, some pathway mutations are not well represented in cell lines, for example the hedgehog pathway, via smoothened (SMO) and GLI1/2/3 mutations do not appear in any known cell line and no cell line is responsive to SMO antagonists (ref: http://www.ncbi.nlm.nih.gov/pubmed/20881279 and unpublished results). Another example are IDH1 and IDH2 gain of function mutations that are commonly found in Glialblastoma and AML in clinical settings, yet very few, one or two, cell lines harbor these activating mutations. Therefore, the new generation of IDH1 inhibitors cannot be tested in standard cell proliferation models.

Two new approaches have capitalized on this idea, instead performing panel based screening directly in mice. These are referred to as “mouse avatars” and mouse “co-clinical trials” and both were recently reviewed by Malaney et al. (2014). The “mouse avatar” approach (also known as “xeno-patient trials”) utilizes patient derived tumor xenograft models (PDTX), which rely on implantation of patient tumor samples into immunocompromised mice for study with pharmacological tool compounds. Tumor samples are genetically characterized both when removed from the patient as well as at intervals after transplantation into mice. While PDTX models have been generated for most major human cancers many challenges still remain for this platform to be widely adopted (cost and engraftment difficulty); however, we anticipate that the approach will continue to grow in prominence.

The second concept of mouse “co-clinical trials” involves using genetically engineered mouse models (GEMMs) in parallel with on-going human clinical trials as a way of anticipating response in the human arm of the trial (Nardella et al., 2011). Several co-clinical trials with GEMMs have been initiated recently with mixed results. In a successful example, Chen et al. (2012) have tested the use of the MEK inhibitor selumetinib in combination with docetaxel in a KRAS-mutant lung cancer trial. This effort led to the observation that the combination therapy outperformed monotherapy in cases where KRAS mutation alone or KRAS and p53 mutation were both present; however, mice with KRAS and LKB1 mutation were resistant to the combination therapy. This data suggests that patients in the clinical trial should be screened for LKB1 mutations in parallel as they may become resistant to therapy. This result, and others like it, could have a significant impact on the human arm of the co-clincial trial and could inform patient stratification for future human trials as well. We anticipate that co-clinical trials will be focused in nature compared to more exploratory cell line panel screens, but could impact the shape of human clinical trials and influence their outcomes while the trial is in progress.

## USING CELLULAR MODELS IN NON-ONCOLOGY FIELDS

Beyond oncology indications, immunological profiling of patient derived blood cells represents a fruitful and relatively straightforward method to profile drug candidates across human genomic variation. From blood draws, peripheral blood mononuclear cells (PBMCs) can be isolated and are commonly used in experiments; large numbers of patient samples can be profiled with this ready source of cells. A key issue to using PBMCs is that they are a mixed cell population representing many of the various immunological lineages, including B and T cells, macrophages, etc. Until recently this represented a significant hurdle because only a few bulk attributes could be used to monitor response to compounds thus the cellular and mechanistic resolution was not nearly high enough. Advances in single cell analytical techniques have changed the landscape and now enable researchers to examine individual cellular responses.

Bodenmiller et al. (2012) recently described a clever proof of concept experiment using a new instrument called a CyTof. The CyTof is essentially a cell sorter that measures mass tags rather than fluorescent tags typical of a FACS instrument. The higher resolution of the mass spectrometer enables a 10–50x improvement in the number of tags that can be read per cell, and this enables identification of each cell type in the sorting along with multiple signaling pathway readouts on a per cell basis. In their study, PBMCs from eight patients were collected and separated into 14 cell types and further sub-division is possible. They simultaneously measured well characterized signaling cascade markers in each cell, like phospho-ERK and phoso-STAT5 in the presence and absence of different immuno stimulatory treatments such as LPS and Interferon-gamma. The data clearly show that cell type specificity is achieved, for example only cell types that express TLR4 respond to the LPS stimulation. What makes the study exceptional is that they then look at the responses across all these condition in the context of about 30 drugs or drug-like tool molecules. The authors generally see similarities between the different patient samples, but do note occasional differences, suggestive of environmental or genetic differences.

While the Bodenmiller study was not powered or designed to explore the genetic or environmental factors that may give rise to these subtleties, it is not a far stretch to imagine hundreds of patients or even longitudinal studies examining *ex vivo* response of PBMCs to various stimuli and drug candidates. Differential response between subjects could be explored in more depth with the goal of correlating and attributing genetic or environment factors (for example allergies that immuno activate cells and immuno inhibitory anti-histamines) to mechanistic changes in cell specific signaling pathways. Additionally, improvements are being made in single cell mRNA detection technologies and these are an attractive alternative to the CyTof technology due to enhanced flexibility of nucleic acid detection technologies (Ståhlberg and Bengtsson, 2010; Shalek et al., 2013). From these types of studies, predictive and stratifying biomarkers could be built and deployed to build clinical trials with better signal to noise ratios.

## iPS DERIVED CELLS

Induced Pluripotent Stem Cell technology has created a massive paradigm shift in disease modeling. Originally described by Takahashi et al. (2007), the introduction of a handful of “reprograming” transcription factors can revert easy to acquire fibroblasts and blood cells to a pluripotent state. These pluripotent cells can then be differentiated into many different cell types and cell structures. Neural progenitors and various types of neurons turn out to be one of the more straight-forward cell types to make and these neuronal models were simply not available to the general research community previously; most researchers use rodent derived primary neurons as a cellular model. Heart, liver, eye, and many other cell types can also be generated. These cells can be grown is a more traditional 2D cell model and also in 3D organ like precursors, including recent reports of cerebral organoids, also known as mini-brains (Lancaster et al., 2013). These multicellular structures are often limited in growth simply by the lack of blood innervation and lack of oxygenation. This is a highly active field of research and we should expect many innovations in the future.

Because iPS use relatively standard cell culture techniques there are already many efforts in academia and commercial ventures to scale up collections. A particularly good and recent review written by McKernan and Watt (2013) details many ongoing large scale collection efforts. Summing all the efforts listed in that review there are currently ~150 cell lines available from public institutions, but there could be more than 20,000 cell lines from 10,000 individuals available in the next 3–5 years. With good data ascertainment for both clinical characterization and genetic profiling, these collections could be enormously valuable.

Most iPS and neuronal disease modeling to date has focused on highly penetrant monogenic diseases. In these experiments researchers collect cells from diseased and normal control patients, reprogram them to iPS states and differentiate them into various neuronal subtypes. Typically characterization of the neurons leads to discovery of a difference in phenotype that reflect the disease state and that, in turn, enables a platform for further characterization and functional screening. Examples here include Pheland–McDermid syndrome (Shcheglovitov et al., 2013), Timothy Syndrome (Paşca et al., 2011; Yazawa et al., 2011; Krey et al., 2012), ALS (Di Giorgio et al., 2008; Dimos et al., 2008), and there are many others. Expanding these collections across large numbers of monogenic diseases offers an opportunity to standardize the QC and neuronal differentiation procedures. Universal assay formats, such as mRNA readouts for cell types or neuronal activity, or activity measures using a system like the MANTRA (Hempel et al., 2011), can be used when pharmacologically profiling. For example, a collection of 250 iPS derived cortical neurons from 25 monogenic diseases (10 lines per disease) could be profiled across hundreds of drugs or drugs candidates using mRNA activity markers. Questions could be asked like what drugs increase the levels of specific synaptic activity markers such as Arc or c-Fos. When contextualized across large numbers of cell lines and compounds, this should reduce potential biases for interpretation and lead to robust results. Although these numbers of iPS cells are considered extremely large for most labs, groups like the New York Stem Cell Foundation (http://www.nyscf.org/) have successfully automated many of the processes associated with culturing stem cells and offer hope on the path to achieving assays scale with iPS derived cells.

One criticism of the this approach is that it is conceptually limited to severe monogenetic disease where penetrance is very high in patients and the expectation is that distinctive iPS derived neuronal phenotypes are related to the disease. Polygenic diseases with high heritability, like schizophrenia, present a much greater challenge. The genetic variants associated with these “common” diseases are thought to exert only a small change to disease risk, and presumably cellular phenotype. Defining what the disease associated variants do at a functional level is a key driver for current research (see commentary by Edwards et al., 2013). Many, if not most, are thought to exert subtle mRNA expression level changes, often confusingly called eQTLs. Because these affects are small and because specific gene regulation can be biased by genome background, hundreds, maybe thousands or tens of thousands of neurons will need to be molecularly and then pharmacologically profiled. Luckily these large scale iPS collections are now coming together. Additionally because common variants are present at rates > 5% throughout the population there is no need to collect harder to get disease patient samples and instead healthy volunteers can be used.

## CONCLUSION

Using large numbers of human cellular models is a proven method to identify patients and the fundamental genetics responsible for disease and drug response. These models have been used for decades to characterize mid to late stage pre-clinical drugs in oncology (e.g., the NCI-60). In the last 5–7 years it has been shown by the CCLE and similar efforts that expanding the numbers of cellular models by 10-fold dramatically improves the resolution of the genetic models and enables discovery of the fundamental biology of tumor growth. Similar strategies can be used for immunological profiling of blood cells or neurological profiling of iPS derived neurons. Scaling up these processes will involve significant investments in infrastructure, methods, and QC to achieve reliable models, especially in the nascent iPS derived neuron field. Improvements in sequencing and genetics technologies are far exceeding the ability to build animal models and we believe that these human models will be more predictive by reflecting the patient diversity presented by people in the clinic.

## Conflict of Interest Statement

The authors declare that the research was conducted in the absence of any commercial or financial relationships that could be construed as a potential conflict of interest.
